# Alterations in the metabolic networks of temporal lobe epilepsy patients: A graph theoretical analysis using FDG-PET

**DOI:** 10.1016/j.nicl.2020.102349

**Published:** 2020-07-15

**Authors:** Hye-Kyung Shim, Ho-Joon Lee, Sung Eun Kim, Byung In Lee, Seongho Park, Kang Min Park

**Affiliations:** a Department of Nuclear Medicine, Haeundae Paik Hospital, Inje University College of Medicine, Busan, Republic of Korea; b Department of Radiology, Haeundae Paik Hospital, Inje University College of Medicine, Busan, Republic of Korea; c Department of Neurology, Haeundae Paik Hospital, Inje University College of Medicine, Busan, Republic of Korea

**Keywords:** Hippocampal sclerosis, Epilepsy, Metabolic connectivity

## Abstract

**Objective:**

The aim of this study is to investigate changes in metabolic networks based on fluorodeoxyglucose positron emission tomography (FDG-PET) in patients with drug-resistant temporal lobe epilepsy (TLE) (with and without hippocampal sclerosis [HS]) when compared with healthy controls.

**Methods:**

We retrospectively enrolled 30 patients with drug-resistant temporal lobe epilepsy (17 patients with HS and 13 patients without HS) and 39 healthy controls. All subjects underwent interictal FDG-PET scans, which were analyzed to obtain metabolic connectivity using graph theoretical analysis. We investigated the differences in metabolic connectivity between patients with drug-resistant TLE (with and without HS) and healthy controls.

**Results:**

When compared with healthy controls, TLE patients with HS showed alterations of global and local metabolic connectivity. When considering global connectivity, TLE patients with HS had a decreased average degree with increased modularity. When considering local connectivity, TLE patients with HS displayed alterations of betweeness centrality in widespread regions. However, there were no alterations of global metabolic connectivity in TLE patients without HS when compared with healthy controls. In addition, when compared to TLE patients without HS, TLE patients with HS had increased modularity.

**Significance:**

Our study demonstrates more severe alterations in metabolic networks based on FDG-PET in TLE patients with HS than in those without HS and healthy controls. This may represent distinct epileptic networks in TLE patients with HS versus those without HS, although both are drug-resistant focal epilepsy.

## Highlights

• The aim of this study is to investigate changes in metabolic networks in patients with drug-resistant TLE.
• TLE patients with HS show alterations of metabolic connectivity compared to those without HS.
• This may represent distinct epileptic networks in TLE patients with HS versus those without HS.

## 1 Introduction

Focal epilepsy, the most common form of epilepsy in adults, is a unifocal or multifocal disorder presenting with seizures involving one hemisphere (Scheffer et al., 2017). The major form of treatment of epilepsy is long-term antiepileptic drug (AED) therapy to which approximately 30% of patients are drug-resistant (Kwan and Brodie, 2000). Mesial temporal lobe epilepsy (TLE) with hippocampal sclerosis (HS) is the most common form of drug-resistant focal epilepsy (DRE) in adult patients and is characterized by decreased hippocampal volume and increased signal intensity on T2-weighted imaging on magnetic resonance imaging (MRI) (Malmgren and Thom, 2012). About 10–40% of patients with drug-resistant focal epilepsy have no clear epileptogenic lesions on MRI. This kind of epilepsy is called focal epilepsy of unknown etiology, also previously called cryptogenic focal epilepsy (Scott et al., 1999, Berg et al., 2003, Von Oertzen et al., 2002); although its detection rate depends on the brain MRI protocol or experience of the MRI readers (Von Oertzen et al., 2002). Recent studies have shown that focal epilepsy involves not only focal lesions but also global brain networks, suggesting that focal epilepsy is a disorder of abnormal brain networks (Chiang and Haneef, 2014, Jin et al., 2015, Besson et al., 2014, Li et al., 2017, Haneef and Chiang, 2014, Bernhardt et al., 2019, Ren et al., 2019).

Connectome analysis using several neuroimaging and electrophysiological modalities has gained much recent interest as a formal framework of network analysis, leading to a paradigm shift and conceptualization of epilepsy (Stacey et al., 2019). The types of connectome analysis include structural and functional connectivity. The functional connectivity usually employs the modalities of electroencephalography (EEG), functional MRI, magnetic encephalography (MEG), or positron emission tomography (PET), whereas the structural connectivity can be analyzed based on diffusion tensor imaging (DTI) (Rossini et al., 2019). The functional connectivity based on fluorodeoxyglucose (FDG)-PET is usually mentioned as metabolic connectivity, revealing a metabolic network of the brain.

Multiple studies on the structural and functional connectivity in patients with focal epilepsy based on DTI, EEG, functional MRI, and MEG have been done. However, only a few studies on the metabolic connectivity based on FDG-PET in patients with drug-resistant TLE have been carried out (Wang et al., 2019, Vanicek et al., 2016). These previous studies have demonstrated a disrupted metabolic network in patients with TLE (Wang et al., 2019, Vanicek et al., 2016). However, no researches have investigated the differences of the metabolic network based on FDG-PET between TLE patients with and without HS. FDG-PET allows us to measure regional cerebral glucose metabolism (Alavi et al., 1986). Analyses of glucose metabolism have been used to explore metabolic connectivity in healthy control subjects (Lee et al., 2008), Alzheimer’s dementia (Morbelli et al., 2012), autism (Horwitz et al., 1988); and obsessive–compulsive disorder (Horwitz et al., 1991); suggesting that metabolic connectivity can be assessed by FDG uptake and that metabolic network can be altered in various neuropsychiatric disorders. In analyzing functional connectivity, functional MRI frequently has been used. However, whereas functional MRI measures a combination of hemodynamic parameters for cerebral blood flow and its volume, thus indirectly reflecting the neuronal activities by using blood oxygen level-dependent signal, FDG-PET can construct metabolic connectivity, directly expressing neuronal energy demands (Smith et al., 2002, Hyder et al., 2013). Furthermore, in general, FDG-PET recordings afford better data with respect to signal-to-noise ratios, variance concentration, and out-of-sample replication than do functional MRI data (Yakushev et al., 2017). Whereas MEG is very expensive and not available in most of the epilepsy centers in the world, FDG-PET is more widely used than MEG, especially for a resective epilepsy surgery. Moreover, the EEG signal has a poor spatial resolution, and it cannot detect the activities of the subcortical brain regions. Thus, analysis of metabolic connectivity based on FDG-PET will be enhanced over that with the other modalities (Yakushev et al., 2017).

The aim of this study is to investigate the changes of metabolic connectivity based on FDG-PET in TLE patients with and without HS compared with healthy controls. We hypothesized that there were differences in alterations of metabolic connectivity based on FDG-PET between TLE patients with and without HS when compared with healthy controls. A previous study with functional MRI has shown that TLE patients without HS are associated with decreased connectivity at the temporal neocortex, whereas TLE patients with HS reveal a different pattern showing a hypersynchronous active hippocampus, with abnormal connectivity extending into the thalamus and ventromedial prefrontal cortex (Vaughan et al., 2016). Another study with diffusion tensor imaging has also demonstrated that HS is associated with marked remodeling of connectome topology in TLE, as opposed to TLE without HS (Bernhardt et al., 2019). Thus, we could hypothesize that there are more alterations in the metabolic connectivity in TLE patients with HS compared to those without HS.

## 2 Materials and methods

### 2.1 Subjects

This study was conducted after approval by the Institutional Review Board at our institution was obtained. This retrospective study was performed at a tertiary hospital (Haeundae Paik Hospital). We initially recruited 61 patients with a clinical diagnosis of drug-resistant epilepsy made with FDG-PET from the epilepsy database of the neurology department. All patients underwent FDG-PET for presurgical evaluation from March 2010 to April 2020. Of the 61 patients with epilepsy, we enrolled 17 TLE patients with HS who met the following inclusion criteria (Malmgren and Thom, 2012): 1) the typical brain MRI features of HS including increased signal intensity on T2-weighted/fluid-attenuated inversion recovery (FLAIR) imaging and decreased hippocampal volumes on visual inspection using a 3.0 T MRI scanner, 2) history of focal seizures consistent with medial TLE semiology and 3) ictal epileptiform discharges originating from the medial temporal lobe in video-EEG monitoring. Patients with any other brain lesions except HS were excluded. We also included 13 TLE patients without HS who met the following criteria (Scheffer et al., 2017): 1) normal brain MRI with visual inspection, 2) history of focal seizures consistent with TLE semiology, and 3) ictal epileptiform discharges clearly originating from the unilateral temporal lobe in video-EEG monitoring. We determined the clinical characteristics, including age, sex, age of seizure onset, duration of epilepsy, and number of AED at the time of PET.

We also enrolled 39 healthy controls, who showed normal neurological findings and had no history of medical, neurological, or psychiatric disease.

### 2.2 FDG-PET data acquisition

All participants fasted for at least 6 h before receiving the injection of 0.07 mCi (2.59 MBq) (Alavi et al., 1986). F‐FDG per body weight intravenously in an awake and resting state with eyes closed. PET image acquisition was started approximately 60 min after the injection (mean time of 59 min with ranges from 47 to 79 min). The mean scanning time after the FDG injection was not different between the TLE patients with and without HS (59 vs. 56 min, *p* = 0.665). The PET scanner was a Siemens Biograph 64 HD PET/CT (Siemens AG, Germany). All participants were scanned once, which lasted about 25 min (3.5 min/bed, 7~8 bed/subjects), and the ^18^F‐FDG‐PET image was reconstructed with ordered-subset expectation maximization (OSEM) iterative reconstruction algorithm in a 168 × 168 matrix with a pixel size of 3.4 mm.

### 2.3 FDG-PET data preprocessing and graph theoretical analysis

PET images were realigned and normalized to the MNI template using both the FSL software and SPM12 based on Matlab. FSL was used for skull stripping, resizing the matrix, realignment, and normalization to MNI template. We then used SPM12 to estimate a deformation field to match an individual scan and write the spatially normalized images. Subsequently, the standardized uptake values (SUV) were extracted for 109 regions of interest (ROIs) based on a Harvard-Oxford atlas using “fslstats” and “fslmaths” tools in the FSL software suite Next, the standardized uptake values ratio (SUVR) were calculated using the following equation: SUVR = SUV_ROI_/SUV_whole cerebellum_ (We confirmed that the mean SUV of the whole cerebellum was not different between the TLE patients with and without HS, *p* = 0.399).

We performed metabolic connectivity analysis with Brain Analysis Using Graph Theory (BRAPH; http://braph.org) (Mijalkov et al., 2017). Graphs were built for each group as a collection of nodes representing brain regions connected by edges corresponding to the connections between them. The nodes were defined using the SUVR from 109 ROIs. The edges were calculated as the partial correlation coefficients between every pair of brain regions while controlling for the effects of age and gender. For each group, a metabolic undirected and weighted connectivity matrix was built. To transform a weighted graph into a binary one, we applied thresholding by fixing the fraction of edges to a density of 50. At these network densities, we noted that all correlations were positive and therefore we did not consider the negative correlations, which were set to zero. To detect metabolic differences between groups in the topology of the global network, we calculated the average degree and strength, characteristic path length, global efficiency, local efficiency, mean clustering coefficient, modularity, assortative coefficient, and the small-worldness index (Mijalkov et al., 2017, Farahani et al., 2019). To assess differences between groups in local network topology, we calculated betweenness centrality (Mijalkov et al., 2017, Farahani et al., 2019). We investigated the alterations of network measures in TLE patients with and without HS compared with healthy controls.

### 2.4 Statistical analyses

The comparative analysis of the factors was performed using the Chi-square test or the Fisher exact test for categorical variables and the Student’s *t*-test or Mann-Whitney test for numerical variables. The categorical variables were presented as frequency and percentage. In the comparison of the network measures, we tested the statistical significance of the differences using nonparametric permutation tests with 1000 permutations with the following methods: The tests are performed by first randomly permuting the subjects from both groups and then calculating the differences in the graph measures between the new randomized groups. By repeating this procedure 1000 times, distribution of between-group differences is obtained. The p-values are then calculated as the fraction of the difference distribution values that exceeded the difference value between the actual groups (Mijalkov et al., 2017). Numerical variables were presented as the mean value ± standard deviation or median value with the range dependent on the normality of distribution. A *p*-value of less than 0.05 indicated statistical significance in all analyses. We did multiple corrections with a false discovery rate when analyzing the local metabolic connectivity changes. All statistical tests were performed using MedCalc® (MedCalc Software version 19.1.3, Ostend, Belgium; https://www.medcalc.org; 2019).

## 3 Results

### 3.1 Clinical characteristics in patients with TLE with HS

Table 1 shows the clinical demographics of patients with drug-resistant temporal lobe epilepsy (with and without HS) and healthy controls at the time of FDG-PET acquisition. Age, male ratio, age of onset, duration of epilepsy, and number of AED were not different between the TLE patients with and without HS. Of the 17 TLE patients with HS, eight patients had right HS, whereas seven patients had left HS. Two patients had bilateral HS. Regarding the visual analysis, the PET images of the 16 patients with TLE with HS showed ipsilateral or bilateral temporal lobes or hemispheric hypometabolism. Only one patient had normal PET images by visual inspection. Of the 13 TLE patients without HS, Seven patients had right TLE, whereas six patients had left TLE. Regarding the visual analysis, hypometabolism was found in the temporal lobe of eight patients, whereas five patients had normal PET images by visual inspection.

**Table 1:** Clinical demographics of temporal lobe epilepsy patients with and without hippocampal sclerosis and healthy controls.

	TLE patients with HS (N = 17)	Healthy controls (N = 39)	*p*-value
*Age, years	37.2 ± 12.5	37.1 ± 4.7	0.936
†Male, N (%)	8 (47.1)	18 (46.1)	0.950
Age of seizure onset, years	18.1 ± 11.5		
Duration of epilepsy, months	191.4 ± 138.7		
Number of AEDs, N (range)	3 (2–7)		

	TLE patients without HS (N = 13)	Healthy controls (N = 39)	*p*-value

*Age, years	37.3 ± 7.1	37.1 ± 4.7	0.894
‡Male, N (%)	4 (40.0)	18 (46.1)	1.000
*Age of seizure onset, years	18.7 ± 7.8		
*Duration of epilepsy, months	226.2 ± 89.2		
§Number of AEDs, N (range)	4 (2–5)		

	TLE patients with HS (N = 17)	TLE patients without HS (N = 13)	*p*-value

*Age, years	37.2 ± 12.5	37.3 ± 7.1	0.988
‡Male, N (%)	8 (47.1)	4 (40.0)	1.000
*Age of seizure onset, years	18.1 ± 11.5	18.7 ± 7.8	0.889
*Duration of epilepsy, months	191.4 ± 138.7	226.2 ± 89.2	0.485
§Number of AEDs, N (range)	3 (2–7)	4 (2–5)	0.154

TLE: temporal lobe epilepsy, HS: hippocampal sclerosis, AEDs: antiepileptic drugs

* Student’s *t*-test

† Chi-square test

‡ Fisher exact test

§ Mann-Whitney test

### 3.2 The differences of the SUVR in TLE patients with and without HS and healthy controls

Compared with healthy controls, TLE patients with HS showed alterations of SUVR in widespread regions, whereas those without HS revealed alterations of SUVR in some regions (78 regions in TLE patients with HS vs. 4 regions in TLE patients without HS) (Suppl. 1). However, there were no significant differences in SUVR between TLE patients with and without HS.

### 3.3 The alterations of metabolic connectivity in TLE patients with HS compared with healthy controls

**Fig. 1:**
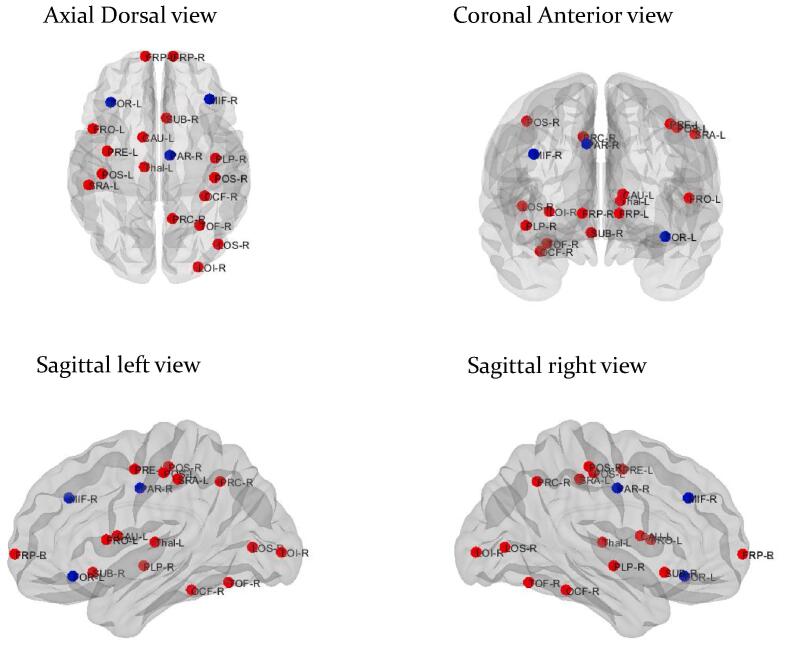
**Alterations of local metabolic connectivity in temporal lobe epilepsy patients with hippocampal sclerosis compared with healthy controls**. The blue circles indicate nodes with decreased betweenness centrality, whereas the red circle reveals node with increased betweenness centrality in temporal lobe epilepsy patients with hippocampal sclerosis compared with healthy controls. CAU-L: left caudate, Thal-L: left thalamus, FRO-L: left frontal operculum, FOR-L: left frontal orbital, FRP-L: left frontal pole, POS-L: left postcentral, PRE-L: left precentral, SRA-L: left supramarginal anterior, FRP-R: right frontal pole, LOI-R: right lateral occipital inferior, LOS-R: right lateral occipital superior, MIF-R: right middle frontal, OCF-R: right occipital fusiform, RAR-R: right paracingulate, PLP-R: right planum temporale, POS-R: right postcentral, PRC-R: right precuneus, SUB-R: right subcallosal, TOF-R: right temporal occipital fusiform. (For interpretation of the references to colour in this figure legend, the reader is referred to the web version of this article.)

Compared with healthy controls, TLE patients with HS showed alterations of global and local connectivity. For global connectivity, TLE patients with HS had decreased average degree with increased modularity when compared with healthy controls (102.844 vs. 107.8532, *p* = 0.033; 0.110 vs. 0.031, *p* = 0.004, respectively) (Table 2). For local connectivity, TLE patients with HS displayed alterations of the betweenness centrality in widespread regions (Fig. 1) (Suppl. 2).

**Table 2:** Alterations of global metabolic connectivity in temporal lobe epilepsy patients with and without hippocampal sclerosis compared with healthy controls.

	TLE patients with HS	Healthy controls	Difference	CI lower	CI upper	*p*-value
Average degree	102.8440	107.853	5.009	−0.133	3.971	*0.033
Average strength	52.4845	59.922	7.437	−14.256	15.877	0.429
Characteristic path length	2.143	1.938	−0.205	−0.553	0.381	0.529
Global efficiency	0.525	0.561	0.036	−0.137	0.125	0.578
Local efficiency	2.958	3.344	0.386	−1.790	1.658	0.633
Mean clustering coefficient	0.463	0.541	0.077	−0.147	0.176	0.494
Modularity	0.110	0.031	−0.079	−0.048	0.017	*0.004
Assortative coefficient	−0.029	−0.013	0.016	−0.011	0.023	0.213
Small-worldness index	0.920	0.974	0.053	−0.022	0.069	0.201

	TLE patients without HS	Healthy controls	Difference	CI lower	CI upper	*p*-value

Average degree	104.935	107.853	2.917	−0.050	15.913	0.757
Average strength	69.290	59.922	−9.368	−20.595	22.315	0.465
Characteristic path length	1.709	1.938	0.228	−0.757	0.534	0.434
Global efficiency	0.666	0.561	−0.104	−0.189	0.156	0.408
Local efficiency	4.749	3.344	−1.405	−2.609	1.833	0.456
Mean clustering coefficient	0.622	0.541	−0.081	−0.192	0.228	0.513
Modularity	0.049	0.031	−0.018	−0.090	0.017	0.908
Assortative coefficient	−0.025	−0.013	0.011	−0.051	0.033	0.655
Small-worldness index	0.930	0.974	0.044	−0.022	0.110	0.847

	TLE patients with HS	TLE patients without HS	Difference	CI lower	CI upper	*p*-value

Average degree	102.844	104.935	2.091	−13.216	3.467	0.227
Average strength	52.484	69.290	16.806	−20.990	20.332	0.199
Characteristic path length	2.143	1.709	−0.434	−0.528	0.616	0.185
Global efficiency	0.525	0.666	0.140	−0.140	0.177	0.211
Local efficiency	2.958	4.749	1.791	−1.708	2.206	0.186
Mean clustering coefficient	0.463	0.622	0.159	−0.190	0.188	0.161
Modularity	0.110	0.049	−0.060	−0.052	0.093	*0.033
Assortative coefficient	−0.029	−0.025	0.004	−0.029	0.065	0.922
Small-worldness index	0.920	0.930	0.009	−0.080	0.042	0.511

CI: 95% confidence interval

* *p* < 0.05

### 3.4 The alterations of metabolic connectivity in TLE patients without HS compared with healthy controls

**Fig. 2:**
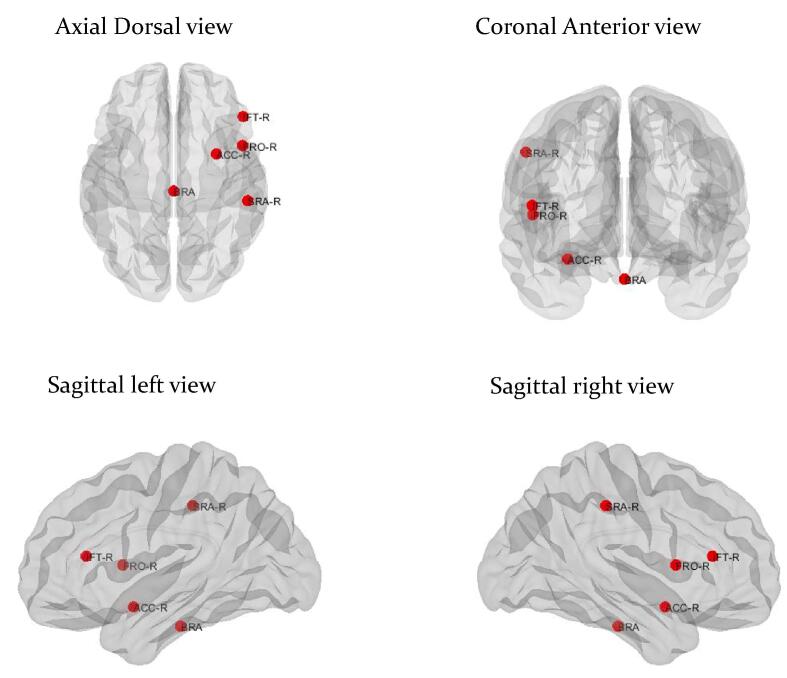
**Alterations of local metabolic connectivity in temporal lobe epilepsy patients without hippocampal sclerosis compared with healthy controls**. The red circle reveals node with increased betweenness centrality in temporal lobe epilepsy patients without hippocampal sclerosis compared with healthy controls. BRA: Brainstem, ACC-R: right accumbens, FRO-R: right frontal operculum, IFT-R: right inferior frontal pars triangularis, SRA-R: right supramarginal anterior. (For interpretation of the references to colour in this figure legend, the reader is referred to the web version of this article.)

There were no alterations of global metabolic connectivity in TLE patients without HS when compared with healthy controls. In addition, with regards to local connectivity, TLE patients without HS showed an alteration of the betweenness centrality in only a few regions (Fig. 2) (Suppl. 2).

### 3.5 The alterations of metabolic connectivity in TLE patients with HS compared with those without HS

**Fig. 3:**
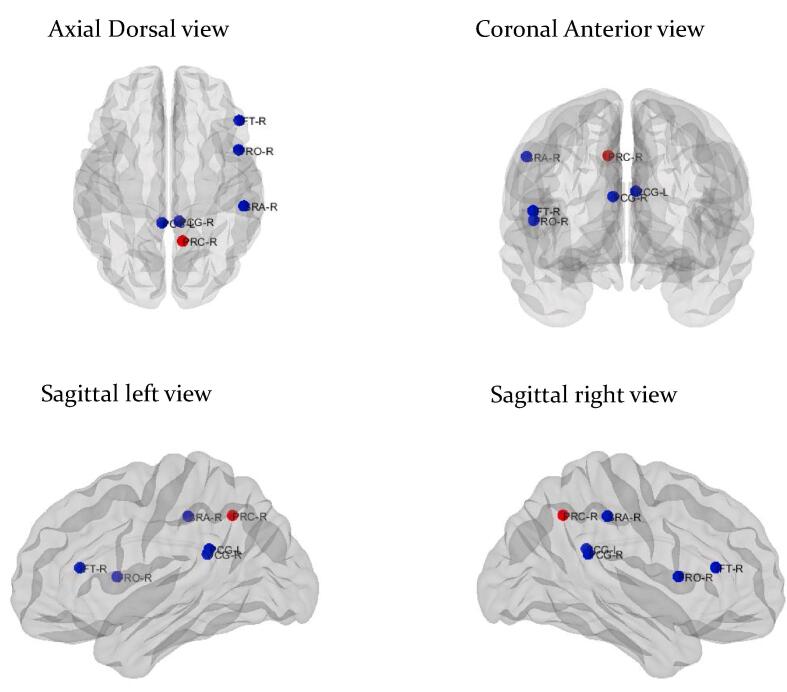
**Alterations of local metabolic connectivity in temporal lobe epilepsy patients with hippocampal sclerosis compared with those without hippocampal sclerosis**. The blue circles indicate nodes with decreased betweenness centrality, whereas the red circle reveals node with increased betweenness centrality in temporal lobe epilepsy patients with hippocampal sclerosis compared with those without hippocampal sclerosis. PCG-L: left posterior cingulate, PCG-R: right posterior cingulate, FRO-R: right frontal operculum, IFT-R: right inferior frontal pars triangularis, PRC-R: right precuneus, SRA-R: right supramarginal anterior. (For interpretation of the references to colour in this figure legend, the reader is referred to the web version of this article.)

When compared with TLE patients without HS, TLE patients with HS showed alterations of global and local connectivity. With regards to global connectivity, TLE patients with HS had increased modularity when compared with those without HS (0.110 vs. 0.049, *p* = 0.033). In addition, with regards to local connectivity, TLE patients with HS showed an alteration of the betweenness centrality in some regions compared to those without HS (Fig. 3) (Suppl. 2).

## 4 Discussion

This study investigated the metabolic network based on FDG-PET in patients with drug-resistant TLE. The main finding of this study was that TLE patients with HS had alterations in metabolic connectivity in the global brain network as well as in the local network when compared with healthy controls and with TLE patients without HS. TLE patients with HS had a decreased average degree with increased modularity in the global network. Additionally, TLE patients with HS had alterations of local metabolic connectivity in the widespread regions. However, we found that there were no alterations of global metabolic networks in TLE patients without HS when compared with healthy controls.

We found that TLE patients with HS had a decreased average degree when compared with healthy controls based on graph theoretical analysis. Graph theory is a mathematical tool that allows for the analysis and quantification of a brain network (Farahani et al., 2019, Gleichgerrcht et al., 2015). It takes into account the full network structure by providing a simple model of the underlying brain connectome, represented by a collection of nodes and edges (Farahani et al., 2019, Gleichgerrcht et al., 2015). By reducing the complex network structure of the brain into a set of parameters that characterize specific topological properties of the network, it enables the study of individual nodes and the network as a whole. This approach has made a considerable impact on recent studies of brain network, especially in fields of epilepsy research (Farahani et al., 2019, Gleichgerrcht et al., 2015). A lower average degree, the total number of edges connected to a node (Englot et al., 2016, Rubinov and Sporns, 2010), implies decreased global brain connectivity in TLE patients with HS. The results of the present study corroborate the concept of epilepsy as a network-level disorder, including focal epilepsy, which traditionally was considered to be a focal brain disorder. It is in agreement with previous researches. Bernhardt *et al* observed markedly increased path length and decreased clustering in TLE patients with HS compared to controls, indicating low structural global network based on diffusion tensor imaging (Bernhardt et al., 2019), and Wang *et al* demonstrated increased characteristic path length with a decrease in global efficiency compared to the controls, indicating impairments in the whole-brain functional network in TLE patients with HS (Wang et al., 2014). Although decreased global connectivity in focal epilepsy was already demonstrated in previous studies, it was not clear whether decreased global connectivity represents the damaging consequences of recurrent seizures or an adaptive mechanism to prevent seizure spread out of the epileptogenic zones (Stacey et al., 2019, Farahani et al., 2019, Englot et al., 2016). Furthermore, we found that TLE patients with HS had increased modularity in global connectivity. Networks with high modularity, a measure of the strength of the division of a network into modules (Stacey et al., 2019, Farahani et al., 2019, Rubinov and Sporns, 2010), have dense connections between the nodes within modules but sparse connections between nodes in different modules. In other words, it reflects the ability of the brain to process specialized functions within highly interconnected metabolic subnetworks (Rubinov and Sporns, 2010). To date, only a limited number of studies have investigated the changes in modularity in TLE patients with HS. Only one study obtained the results of network metrics in two TLE patients with HS while TLE patients with psychosis showed a lower global efficiency, small-worldness, and modularity when compared with TLE patients without psychosis (Sone et al., 2016). Recently, we found that the modularity of the intrinsic thalamic network in patients with juvenile myoclonic epilepsy was significantly higher than in healthy controls (Lee et al., 2019). We can speculate that increased segregation with high metabolic modularity in TLE patients with HS is associated with highly efficient synchronization of the global brain network, which also makes the brain network more prone to seizures. Thus, it could be related to epileptogenic networks in TLE patients with HS.

However, we found that there were no alterations in global metabolic connectivity in TLE patients without HS when compared with healthy controls. In addition, there were significant differences in metabolic connectivity between TLE patients with and without HS. It may represent distinct epileptic networks in TLE patients with HS versus those without HS, although both are drug-resistant focal epilepsy. It could be related to AED response and cognition in patients with epilepsy. In adults, focal epilepsy is usually more difficult to treat than genetic generalized epilepsy (Semah et al., 1998). Among the different types of focal epilepsy, the rate of seizure control is higher in patients with focal epilepsy of unknown etiology (45%) than in TLE patients with HS (11%) (Semah et al., 1998). In children with newly diagnosed epilepsy, neuroimaging abnormality is the single important predictor of enduring medical intractability (Wirrell et al., 2013). Furthermore, Choi and her colleague examined the seizure trajectories of patients with epilepsy developing DRE and identified predictors of seizure trajectory outcomes (Choi et al., 2016). They found that even in the adult patients who failed two AEDs therapy, about one-third of them can achieve early or delayed seizure freedom during medical management, and the patients with normal MRI had a more likelihood of becoming seizure-free than those with MRI abnormalities (Choi et al., 2016). Thus, more alterations of metabolic network in TLE patients with HS could produce a decrease of AED response. Furthermore, when investigating neuroimaging and cognitive function in patients with newly diagnosed focal epilepsy, patients with an abnormal MRI usually have a higher chance of abnormal cognition than those with a normal MRI (Vecchi et al., 2016). We can assume that the higher decrease of AED response and cognitive impairments seen in patients with TLE with HS compared to those without HS might be related to more disruptions of metabolic connectivity.

This is the first study to demonstrate the differences in metabolic connectivity based on FDG-PET between TLE patients with and without HS. However, there were several limitations to this study. First, the sample size was relatively small for this study. However, we could detect statistically significant differences in metabolic connectivity in TLE patients with HS compared with healthy controls, even with this small sample size. Second, there was a selection bias when enrolling patients with drug-resistant focal epilepsy. All of the FDG-PET imaging done in subjects were conducted during presurgical evaluation, thus, the subjects could not represent patients with typical drug-resistant focal epilepsy. Third, this study has a cross-sectional retrospective design and was conducted in a single center. Further prospective multicenter studies with a large sample size are needed to confirm our findings. Lastly, there are several inherent limitations to a PET covariance analysis. It can only be done at the group level, and highly relies on the definition of a homogenous group of subjects. Also, it has a low image resolution and partial volume problems (Veronese et al., 2019).

## 5 Conclusion

Our study successfully demonstrates more severe alterations of metabolic networks based on FDG-PET in TLE patients with HS than in those without HS. It may represent distinct epileptic networks in TLE patients with HS versus those without HS, although both are drug-resistant focal epilepsy.

## CRediT authorship contribution statement

**Hye-Kyung Shim:** Writing - original draft. **Ho-Joon Lee:** Data curation, Software. **Sung Eun Kim:** Writing - review & editing. **Byung In Lee:** Writing - review & editing. **Seongho Park:** Writing - review & editing. **Kang Min Park:** Conceptualization, Methodology.

## Declaration of Competing Interest

The authors declare that they have no known competing financial interests or personal relationships that could have appeared to influence the work reported in this paper.
