# Supplementary material for: Alterations in the metabolic networks of temporal lobe epilepsy patients: A graph theoretical analysis using FDG-PET
Source: Neuroimage Clin. 2020 Jul 15;27:102349. doi: 10.1016/j.nicl.2020.102349 (PMC7374556; doi:10.1016/j.nicl.2020.102349)
Supplement: Supplementary data 1 [file mmc1.docx]

Suppl. 1. The standardized uptake values ratio of regions of interest using a Harvard-Oxford atlas (Nodes) in the temporal lobe epilepsy patients with and without hippocampal sclerosis and healthy controls

|  | TLE patients with HS | | TLE patients without HS | | Healthy Controls | |
| --- | --- | --- | --- | --- | --- | --- |
|  | Mean | SD | Mean | SD | Mean | SD |
| Brainstem | 0.769 | 0.062 | 0.755 | 0.039 | 0.769 | 0.044 |
| Lt. accumbens | 1.272 | 0.144 | 1.315 | 0.099 | 1.361 | 0.130 |
| Lt. amygdala | 0.856 | 0.090 | 0.868 | 0.108 | 0.866 | 0.055 |
| Lt. angular***** | 1.205 | 0.148 | 1.258 | 0.157 | 1.398 | 0.097 |
| Lt. anterior cingulate and paracingulate***** | 1.110 | 0.126 | 1.219 | 0.152 | 1.252 | 0.090 |
| Lt. caudate***** | 1.170 | 0.180 | 1.207 | 0.188 | 1.334 | 0.118 |
| Lt. cuneus | 1.231 | 0.168 | 1.311 | 0.210 | 1.340 | 0.130 |
| Lt. frontal medial***** | 1.256 | 0.165 | 1.349 | 0.160 | 1.446 | 0.117 |
| Lt. frontal operculum***** | 1.239 | 0.153 | 1.350 | 0.169 | 1.492 | 0.133 |
| Lt. frontal orbital***** | 1.117 | 0.113 | 1.215 | 0.148 | 1.300 | 0.118 |
| Lt. frontal pole***** | 1.147 | 0.145 | 1.237 | 0.189 | 1.382 | 0.126 |
| Lt. heschls*****† | 1.343 | 0.225 | 1.383 | 0.142 | 1.556 | 0.125 |
| Lt. hippocampus***** | 0.894 | 0.139 | 0.968 | 0.053 | 0.995 | 0.065 |
| Lt. inferior frontal pars opercularis***** | 1.213 | 0.140 | 1.321 | 0.175 | 1.454 | 0.101 |
| Lt. inferior frontal pars triangularis***** | 1.208 | 0.142 | 1.285 | 0.170 | 1.418 | 0.114 |
| Lt. inferior temporal anterior***** | 0.997 | 0.167 | 1.085 | 0.146 | 1.193 | 0.092 |
| Lt. inferior temporal posterior***** | 1.086 | 0.137 | 1.172 | 0.131 | 1.270 | 0.093 |
| Lt. inferior temporal temporooccipital***** | 1.115 | 0.151 | 1.194 | 0.143 | 1.284 | 0.092 |
| Lt. insular***** | 1.058 | 0.117 | 1.161 | 0.103 | 1.222 | 0.080 |
| Lt. intracalcarine | 1.403 | 0.142 | 1.426 | 0.146 | 1.490 | 0.149 |
| Lt. lateral occipital inferior | 1.103 | 0.170 | 1.192 | 0.166 | 1.219 | 0.093 |
| Lt. lateral occipital superior***** | 1.098 | 0.150 | 1.209 | 0.184 | 1.293 | 0.099 |
| Lt. lingual | 1.193 | 0.136 | 1.270 | 0.128 | 1.299 | 0.086 |
| Lt. middle frontal*****† | 1.274 | 0.143 | 1.345 | 0.168 | 1.514 | 0.113 |
| Lt. middle temporal anterior***** | 1.004 | 0.148 | 1.177 | 0.155 | 1.270 | 0.101 |
| Lt. middle temporal posterior***** | 1.079 | 0.146 | 1.212 | 0.176 | 1.312 | 0.094 |
| Lt. middle temporal temporooccipital***** | 1.113 | 0.144 | 1.191 | 0.162 | 1.286 | 0.092 |
| Lt. occipital fusiform | 1.260 | 0.172 | 1.279 | 0.116 | 1.348 | 0.100 |
| Lt. occipital pole | 1.035 | 0.220 | 1.132 | 0.224 | 1.166 | 0.178 |
| Lt. pallidum | 0.954 | 0.090 | 0.970 | 0.083 | 1.002 | 0.085 |
| Lt. paracingulate***** | 1.230 | 0.134 | 1.342 | 0.162 | 1.454 | 0.103 |
| Lt. parahippocampal anterior | 0.866 | 0.076 | 0.919 | 0.084 | 0.942 | 0.069 |
| Lt. parahippocampal posterior***** | 0.887 | 0.097 | 0.969 | 0.111 | 0.997 | 0.073 |
| Lt. parietal operculum***** | 1.235 | 0.161 | 1.342 | 0.144 | 1.391 | 0.115 |
| Lt. planum polare***** | 0.936 | 0.119 | 1.006 | 0.098 | 1.060 | 0.084 |
| Lt. planum temporale | 1.207 | 0.157 | 1.282 | 0.162 | 1.337 | 0.135 |
| Lt. postcentral***** | 1.088 | 0.118 | 1.206 | 0.150 | 1.273 | 0.100 |
| Lt. posterior cingulate***** | 1.277 | 0.127 | 1.329 | 0.138 | 1.465 | 0.123 |
| Lt. precentral***** | 1.171 | 0.113 | 1.268 | 0.139 | 1.345 | 0.092 |
| Lt. precuneus***** | 1.293 | 0.163 | 1.373 | 0.164 | 1.484 | 0.108 |
| Lt. putamen | 1.459 | 0.130 | 1.492 | 0.096 | 1.568 | 0.111 |
| Lt. rolandic operculum***** | 1.165 | 0.128 | 1.278 | 0.120 | 1.355 | 0.101 |
| Lt._subcallosal | 1.088 | 0.116 | 1.149 | 0.116 | 1.162 | 0.089 |
| Lt. superior frontal***** | 1.216 | 0.134 | 1.302 | 0.151 | 1.458 | 0.119 |
| Lt. superior parietal***** | 1.115 | 0.116 | 1.234 | 0.162 | 1.305 | 0.116 |
| Lt. superior temporal anterior***** | 1.048 | 0.145 | 1.174 | 0.132 | 1.234 | 0.092 |
| Lt. superior temporal posterior***** | 1.131 | 0.165 | 1.242 | 0.157 | 1.354 | 0.120 |
| Lt. supracalcarine | 1.423 | 0.191 | 1.441 | 0.177 | 1.557 | 0.119 |
| Lt. supramarginal anterior***** | 1.073 | 0.111 | 1.185 | 0.183 | 1.280 | 0.112 |
| Lt. supramarginal posterior***** | 1.121 | 0.127 | 1.217 | 0.186 | 1.327 | 0.098 |
| Lt. temporal fusiform anterior | 0.956 | 0.119 | 0.998 | 0.089 | 1.054 | 0.081 |
| Lt. temporal fusiform posterior***** | 1.045 | 0.105 | 1.120 | 0.121 | 1.163 | 0.081 |
| Lt. temporal occipital fusiform | 1.207 | 0.151 | 1.250 | 0.116 | 1.315 | 0.092 |
| Lt. temporal pole***** | 0.855 | 0.107 | 0.969 | 0.120 | 1.008 | 0.067 |
| Lt. thalamus | 1.056 | 0.091 | 1.075 | 0.082 | 1.134 | 0.076 |
| Rt. accumbens | 1.228 | 0.106 | 1.226 | 0.063 | 1.285 | 0.116 |
| Rt. amygdala | 0.837 | 0.107 | 0.858 | 0.105 | 0.864 | 0.055 |
| Rt. angular***** | 1.164 | 0.084 | 1.230 | 0.151 | 1.350 | 0.089 |
| Rt. anterior cingulate and paracingulate***** | 1.113 | 0.127 | 1.208 | 0.135 | 1.243 | 0.082 |
| Rt. caudate***** | 1.179 | 0.146 | 1.207 | 0.205 | 1.352 | 0.126 |
| Rt. cuneus | 1.280 | 0.157 | 1.323 | 0.205 | 1.367 | 0.130 |
| Rt. frontal medial***** | 1.251 | 0.137 | 1.325 | 0.155 | 1.413 | 0.110 |
| Rt. frontal operculum***** | 1.278 | 0.118 | 1.363 | 0.191 | 1.513 | 0.153 |
| Rt. frontal orbital***** | 1.112 | 0.107 | 1.226 | 0.132 | 1.280 | 0.114 |
| Rt. frontal pole***** | 1.164 | 0.102 | 1.249 | 0.178 | 1.385 | 0.120 |
| Rt. heschls*****† | 1.325 | 0.134 | 1.372 | 0.103 | 1.549 | 0.139 |
| Rt. hippocampus***** | 0.866 | 0.128 | 0.934 | 0.098 | 0.985 | 0.061 |
| Rt. inferior frontal pars opercularis***** | 1.276 | 0.101 | 1.329 | 0.166 | 1.484 | 0.129 |
| Rt. inferior frontal pars triangularis***** | 1.181 | 0.108 | 1.264 | 0.190 | 1.401 | 0.133 |
| Rt. inferior temporal anterior***** | 0.988 | 0.107 | 1.107 | 0.151 | 1.177 | 0.092 |
| Rt. inferior temporal posterior***** | 1.077 | 0.109 | 1.157 | 0.145 | 1.237 | 0.087 |
| Rt. inferior temporal temporooccipital***** | 1.090 | 0.091 | 1.165 | 0.137 | 1.263 | 0.087 |
| Rt. insular***** | 1.064 | 0.093 | 1.141 | 0.108 | 1.218 | 0.079 |
| Rt. intracalcarine | 1.376 | 0.156 | 1.424 | 0.119 | 1.477 | 0.133 |
| Rt. lateral occipital inferior***** | 1.140 | 0.114 | 1.215 | 0.179 | 1.259 | 0.083 |
| Rt. lateral occipital superior***** | 1.118 | 0.118 | 1.207 | 0.181 | 1.281 | 0.097 |
| Rt. lingual | 1.164 | 0.105 | 1.250 | 0.140 | 1.278 | 0.093 |
| Rt. middle frontal***** | 1.282 | 0.088 | 1.362 | 0.163 | 1.528 | 0.120 |
| Rt. middle temporal anterior***** | 1.012 | 0.127 | 1.147 | 0.175 | 1.241 | 0.111 |
| Rt. middle temporal posterior***** | 1.091 | 0.106 | 1.195 | 0.167 | 1.317 | 0.101 |
| Rt. middle temporal temporooccipital***** | 1.120 | 0.088 | 1.192 | 0.146 | 1.277 | 0.090 |
| Rt. occipital fusiform | 1.264 | 0.149 | 1.284 | 0.127 | 1.351 | 0.095 |
| Rt. occipital pole | 1.045 | 0.201 | 1.140 | 0.231 | 1.203 | 0.151 |
| Rt. pallidum | 1.000 | 0.090 | 0.996 | 0.071 | 1.054 | 0.101 |
| Rt. paracingulate***** | 1.227 | 0.120 | 1.342 | 0.186 | 1.465 | 0.111 |
| Rt. parahippocampal anterior***** | 0.824 | 0.113 | 0.897 | 0.108 | 0.923 | 0.061 |
| Rt. parahippocampal posterior | 0.895 | 0.103 | 0.953 | 0.115 | 0.976 | 0.063 |
| Rt. parietal operculum***** | 1.219 | 0.149 | 1.282 | 0.150 | 1.373 | 0.128 |
| Rt. planum polare***** | 0.957 | 0.121 | 1.032 | 0.108 | 1.092 | 0.083 |
| Rt. planum temporale***** | 1.181 | 0.134 | 1.268 | 0.146 | 1.340 | 0.124 |
| Rt. postcentral***** | 1.111 | 0.100 | 1.212 | 0.137 | 1.276 | 0.094 |
| Rt. posterior cingulate***** | 1.268 | 0.076 | 1.321 | 0.140 | 1.449 | 0.117 |
| Rt. precentral***** | 1.182 | 0.091 | 1.274 | 0.145 | 1.347 | 0.095 |
| Rt. precuneus***** | 1.273 | 0.122 | 1.356 | 0.163 | 1.460 | 0.103 |
| Rt. putamen | 1.466 | 0.135 | 1.514 | 0.100 | 1.569 | 0.111 |
| Rt. rolandic operculum***** | 1.206 | 0.127 | 1.276 | 0.124 | 1.374 | 0.087 |
| Rt. subcallosal | 1.091 | 0.119 | 1.156 | 0.113 | 1.175 | 0.084 |
| Rt. superior frontal***** | 1.202 | 0.100 | 1.291 | 0.162 | 1.441 | 0.115 |
| Rt. superior parietal***** | 1.107 | 0.106 | 1.212 | 0.175 | 1.267 | 0.114 |
| Rt. superior temporal anterior***** | 1.049 | 0.148 | 1.153 | 0.120 | 1.256 | 0.088 |
| Rt. superior temporal posterior*****† | 1.148 | 0.121 | 1.245 | 0.120 | 1.410 | 0.115 |
| Rt. supracalcarine | 1.392 | 0.223 | 1.475 | 0.173 | 1.526 | 0.121 |
| Rt. supramarginal anterior***** | 1.107 | 0.086 | 1.190 | 0.151 | 1.290 | 0.096 |
| Rt. supramarginal posterior***** | 1.149 | 0.078 | 1.202 | 0.146 | 1.347 | 0.107 |
| Rt. temporal fusiform anterior | 0.941 | 0.127 | 0.980 | 0.119 | 1.038 | 0.075 |
| Rt. temporal fusiform posterior***** | 1.003 | 0.117 | 1.074 | 0.157 | 1.139 | 0.083 |
| Rt. temporal occipital fusiform***** | 1.179 | 0.109 | 1.231 | 0.114 | 1.303 | 0.089 |
| Rt. temporal pole***** | 0.864 | 0.120 | 0.977 | 0.132 | 1.014 | 0.070 |
| Rt. thalamus***** | 1.040 | 0.087 | 1.073 | 0.104 | 1.125 | 0.073 |

TLE: temporal lobe epilepsy, HS: hippocampal sclerosis, SD: standard deviation

*****Significantly different regions between temporal lobe epilepsy patients with hippocampal sclerosis and healthy controls

† Significantly different regions between temporal lobe epilepsy patients without hippocampal sclerosis and healthy controls
