# Supplementary material for: Alterations in the metabolic networks of temporal lobe epilepsy patients: A graph theoretical analysis using FDG-PET
Source: Neuroimage Clin. 2020 Jul 15;27:102349. doi: 10.1016/j.nicl.2020.102349 (PMC7374556; doi:10.1016/j.nicl.2020.102349)
Supplement: Supplementary data 2 [file mmc2.docx]

Suppl. 2. Alterations of the local metabolic connectivity among temporal lobe epilepsy patients with and without hippocampal sclerosis and healthy controls

|  | TLE patients with HS | Healthy controls | Difference | CI lower | CI upper | *p*-value |
| --- | --- | --- | --- | --- | --- | --- |
| Brainstem | 0.000 | 0.000 | 0.000 | 0.000 | 0.000 | 0.050 |
| Lt. accumbens | 0.001 | 0.000 | -0.001 | -0.006 | 0.001 | 0.826 |
| Lt. amygdala | 0.001 | 0.002 | 0.001 | -0.007 | 0.002 | 0.368 |
| Lt. caudate | 0.007 | 0.000 | -0.007 | -0.004 | 0.000 | 0.006 |
| Lt. hippocampus | 0.000 | 0.000 | 0.000 | -0.005 | 0.001 | 0.201 |
| Lt. pallidum | 0.002 | 0.000 | -0.002 | -0.005 | 0.002 | 0.326 |
| Lt. putamen | 0.005 | 0.001 | -0.004 | -0.006 | 0.003 | 0.157 |
| Lt. thalamus | 0.004 | 0.000 | -0.004 | -0.004 | 0.001 | 0.049 |
| Rt. accumbens | 0.000 | 0.000 | 0.000 | -0.001 | 0.000 | 0.877 |
| Rt. amygdala | 0.001 | 0.000 | 0.000 | -0.002 | 0.001 | 0.868 |
| Rt. caudate | 0.002 | 0.000 | -0.002 | -0.003 | 0.000 | 0.121 |
| Rt. hippocampus | 0.000 | 0.000 | 0.000 | -0.005 | 0.001 | 0.469 |
| Rt. pallidum | 0.000 | 0.000 | 0.000 | -0.002 | 0.000 | 0.221 |
| Rt. putamen | 0.001 | 0.000 | -0.001 | -0.009 | 0.006 | 0.966 |
| Rt. thalamus | 0.002 | 0.000 | -0.002 | -0.004 | 0.001 | 0.128 |
| Lt. angular | 0.000 | 0.000 | 0.000 | -0.002 | 0.000 | 0.208 |
| Lt. rolandic operculum | 0.005 | 0.000 | -0.005 | -0.007 | 0.002 | 0.108 |
| Lt. anterior cingulate and paracingulate | 0.000 | 0.001 | 0.001 | -0.009 | 0.002 | 0.249 |
| Lt. posterior cingulate | 0.000 | 0.000 | 0.000 | -0.002 | 0.000 | 0.133 |
| Lt. cuneus | 0.000 | 0.000 | 0.000 | -0.003 | 0.001 | 0.342 |
| Lt. frontal medial | 0.006 | 0.000 | -0.006 | -0.006 | 0.001 | 0.060 |
| Lt. frontal operculum | 0.008 | 0.001 | -0.007 | -0.004 | 0.001 | 0.016 |
| Lt. frontal orbital | 0.001 | 0.006 | 0.005 | -0.005 | 0.001 | 0.022 |
| Lt. frontal pole | 0.004 | 0.000 | -0.004 | -0.004 | 0.000 | 0.043 |
| Lt. heschls | 0.008 | 0.003 | -0.005 | -0.011 | 0.006 | 0.416 |
| Lt. inferior frontal pars opercularis | 0.003 | 0.005 | 0.001 | -0.010 | 0.003 | 0.348 |
| Lt. inferior frontal pars triangularis | 0.000 | 0.002 | 0.002 | -0.007 | 0.001 | 0.099 |
| Lt. inferior temporal anterior | 0.000 | 0.000 | 0.000 | -0.003 | 0.001 | 0.527 |
| Lt. inferior temporal posterior | 0.000 | 0.002 | 0.002 | -0.007 | 0.002 | 0.195 |
| Lt. inferior temporal temporooccipital | 0.000 | 0.000 | 0.000 | -0.005 | 0.000 | 0.158 |
| Lt. insular | 0.005 | 0.006 | 0.001 | -0.009 | 0.004 | 0.532 |
| Lt. intracalcarine | 0.000 | 0.000 | 0.000 | -0.001 | 0.000 | 0.114 |
| Lt. lateral occipital inferior | 0.000 | 0.003 | 0.003 | -0.007 | 0.004 | 0.155 |
| Lt. lateral occipital superior | 0.001 | 0.001 | 0.000 | -0.005 | 0.002 | 0.907 |
| Lt. lingual | 0.001 | 0.000 | 0.000 | -0.006 | 0.001 | 0.653 |
| Lt. middle frontal | 0.003 | 0.004 | 0.001 | -0.005 | 0.003 | 0.675 |
| Lt. middle temporal anterior | 0.000 | 0.000 | 0.000 | -0.007 | 0.001 | 0.640 |
| Lt. middle temporal posterior | 0.000 | 0.001 | 0.001 | -0.003 | 0.001 | 0.224 |
| Lt. middle temporal temporooccipital | 0.000 | 0.000 | 0.000 | -0.009 | 0.001 | 0.579 |
| Lt. occipital fusiform | 0.003 | 0.000 | -0.003 | -0.004 | 0.001 | 0.061 |
| Lt. occipital pole | 0.000 | 0.000 | 0.000 | -0.001 | 0.000 | 0.093 |
| Lt. paracingulate | 0.014 | 0.006 | -0.008 | -0.011 | 0.005 | 0.104 |
| Lt. parahippocampal anterior | 0.001 | 0.000 | 0.000 | -0.007 | 0.002 | 0.806 |
| Lt. parahippocampal posterior | 0.000 | 0.000 | 0.000 | -0.001 | 0.000 | 0.382 |
| Lt. parietal operculum | 0.000 | 0.000 | 0.000 | -0.003 | 0.000 | 0.924 |
| Lt. planum polare | 0.006 | 0.001 | -0.005 | -0.007 | 0.004 | 0.136 |
| Lt. planum temporale | 0.000 | 0.000 | 0.000 | -0.003 | 0.000 | 0.686 |
| Lt. postcentral | 0.009 | 0.001 | -0.009 | -0.006 | 0.002 | 0.024 |
| Lt. precentral | 0.011 | 0.001 | -0.011 | -0.007 | 0.004 | 0.020 |
| Lt. precuneuous | 0.003 | 0.003 | 0.000 | -0.007 | 0.003 | 0.652 |
| Lt. subcallosal | 0.006 | 0.000 | -0.006 | -0.007 | 0.003 | 0.069 |
| Lt. superior frontal | 0.006 | 0.002 | -0.004 | -0.006 | 0.001 | 0.107 |
| Lt. superior parietal | 0.002 | 0.000 | -0.002 | -0.004 | 0.000 | 0.105 |
| Lt. superior temporal anterior | 0.000 | 0.000 | 0.000 | -0.002 | 0.000 | 0.956 |
| Lt. superior temporal posterior | 0.000 | 0.000 | 0.000 | -0.002 | 0.000 | 0.125 |
| Lt. supracalcarine | 0.001 | 0.001 | 0.000 | -0.003 | 0.001 | 0.487 |
| Lt. supramarginal anterior | 0.005 | 0.000 | -0.005 | -0.002 | 0.000 | 0.004 |
| Lt. supramarginal posterior | 0.000 | 0.000 | 0.000 | -0.003 | 0.000 | 0.343 |
| Lt. temporal fusiform anterior | 0.001 | 0.000 | -0.001 | -0.006 | 0.003 | 0.774 |
| Lt. temporal fusiform posterior | 0.001 | 0.000 | -0.001 | -0.008 | 0.002 | 0.851 |
| Lt. temporal occipital fusiform | 0.001 | 0.001 | -0.001 | -0.005 | 0.000 | 0.970 |
| Lt. temporal pole | 0.000 | 0.000 | 0.000 | -0.005 | 0.002 | 0.627 |
| Rt. angular | 0.001 | 0.000 | -0.001 | -0.001 | 0.000 | 0.074 |
| Rt. rolandic operculum | 0.001 | 0.001 | 0.000 | -0.006 | 0.005 | 0.870 |
| Rt. anterior cingulate and paracingulate | 0.001 | 0.000 | -0.001 | -0.007 | 0.002 | 0.806 |
| Rt. posterior cingulate | 0.000 | 0.000 | 0.000 | -0.002 | 0.000 | 0.206 |
| Rt. cuneus | 0.001 | 0.000 | -0.001 | -0.002 | 0.000 | 0.805 |
| Rt. frontal medial | 0.005 | 0.001 | -0.004 | -0.006 | 0.002 | 0.133 |
| Rt. frontal operculum | 0.001 | 0.000 | -0.001 | -0.002 | 0.000 | 0.866 |
| Rt. frontal orbital | 0.003 | 0.000 | -0.003 | -0.003 | 0.000 | 0.056 |
| Rt. frontal pole | 0.003 | 0.000 | -0.003 | -0.002 | 0.000 | 0.026 |
| Rt. heschls | 0.006 | 0.000 | -0.006 | -0.008 | 0.005 | 0.137 |
| Rt. inferior frontal pars opercularis | 0.001 | 0.000 | -0.001 | -0.002 | 0.000 | 0.882 |
| Rt. inferior frontal pars triangularis | 0.000 | 0.000 | 0.000 | -0.004 | 0.001 | 0.223 |
| Rt. inferior temporal anterior | 0.004 | 0.000 | -0.003 | -0.009 | 0.003 | 0.531 |
| Rt. inferior temporal posterior | 0.000 | 0.001 | 0.001 | -0.005 | 0.002 | 0.359 |
| Rt. inferior temporal temporooccipital | 0.001 | 0.000 | -0.001 | -0.002 | 0.000 | 0.080 |
| Rt. insular | 0.012 | 0.007 | -0.005 | -0.017 | 0.008 | 0.603 |
| Rt. intracalcarine | 0.000 | 0.000 | 0.000 | -0.003 | 0.000 | 0.182 |
| Rt. lateral occipital inferior | 0.018 | 0.000 | -0.018 | -0.007 | 0.002 | 0.001 |
| Rt. lateral occipital superior | 0.010 | 0.000 | -0.010 | -0.002 | 0.000 | 0.001 |
| Rt. lingual | 0.001 | 0.001 | -0.001 | -0.008 | 0.003 | 0.816 |
| Rt. middle frontal | 0.000 | 0.003 | 0.003 | -0.003 | 0.001 | 0.027 |
| Rt. middle temporal anterior | 0.000 | 0.000 | 0.000 | -0.004 | 0.001 | 0.698 |
| Rt. middle temporal posterior | 0.002 | 0.008 | 0.007 | -0.008 | 0.006 | 0.104 |
| Rt. middle temporal temporooccipital | 0.001 | 0.000 | -0.001 | -0.005 | 0.001 | 0.855 |
| Rt. occipital fusiform | 0.013 | 0.001 | -0.013 | -0.009 | 0.009 | 0.026 |
| Rt. occipital pole | 0.001 | 0.001 | 0.000 | -0.007 | 0.001 | 0.331 |
| Rt. paracingulate | 0.001 | 0.006 | 0.005 | -0.006 | 0.002 | 0.049 |
| Rt. parahippocampal anterior | 0.000 | 0.000 | 0.000 | -0.003 | 0.000 | 0.138 |
| Rt. parahippocampal posterior | 0.000 | 0.000 | 0.000 | -0.001 | 0.000 | 0.164 |
| Rt. parietal operculum | 0.000 | 0.000 | 0.000 | -0.004 | 0.000 | 0.246 |
| Rt. planum polare | 0.009 | 0.000 | -0.008 | -0.007 | 0.002 | 0.045 |
| Rt. planum temporale | 0.000 | 0.000 | 0.000 | -0.004 | 0.001 | 0.490 |
| Rt. postcentral | 0.011 | 0.001 | -0.010 | -0.005 | 0.004 | 0.010 |
| Rt. precentral | 0.004 | 0.007 | 0.003 | -0.008 | 0.007 | 0.430 |
| Rt. precuneus | 0.012 | 0.001 | -0.012 | -0.005 | 0.003 | 0.006 |
| Rt. subcallosal | 0.008 | 0.000 | -0.008 | -0.005 | 0.003 | 0.021 |
| Rt. superior frontal | 0.003 | 0.002 | -0.001 | -0.005 | 0.001 | 0.892 |
| Rt. superior parietal | 0.000 | 0.000 | 0.000 | -0.002 | 0.000 | 0.944 |
| Rt. superior temporal anterior | 0.001 | 0.000 | -0.001 | -0.002 | 0.000 | 0.776 |
| Rt. superior temporal posterior | 0.000 | 0.000 | 0.000 | -0.004 | 0.000 | 0.219 |
| Rt. supracalcarine | 0.000 | 0.000 | 0.000 | -0.002 | 0.000 | 0.149 |
| Rt. supramargianl anterior | 0.000 | 0.000 | 0.000 | -0.001 | 0.000 | 0.095 |
| Rt. supramargianl posterior | 0.000 | 0.000 | 0.000 | -0.001 | 0.000 | 0.133 |
| Rt. temporal fusiform anterior | 0.001 | 0.000 | -0.001 | -0.004 | 0.001 | 0.702 |
| Rt. temporal fusiform posterior | 0.002 | 0.001 | -0.002 | -0.008 | 0.007 | 0.587 |
| Rt. temporal occipital fusiform | 0.015 | 0.000 | -0.015 | -0.006 | 0.004 | 0.003 |
| Rt. temporal pole | 0.006 | 0.001 | -0.005 | -0.007 | 0.006 | 0.175 |
|  | TLE patients without HS | Healthy controls | Difference | CI lower | CI upper | *p*-value |
| Brainstem | 0.010 | 0.000 | -0.010 | -0.005 | 0.000 | 0.016 |
| Lt. accumbens | 0.000 | 0.000 | 0.000 | -0.006 | 0.000 | 0.163 |
| Lt. amygdala | 0.000 | 0.002 | 0.002 | -0.006 | 0.000 | 0.084 |
| Lt. caudate | 0.000 | 0.000 | 0.000 | -0.006 | 0.000 | 0.141 |
| Lt. hippocampus | 0.000 | 0.000 | 0.000 | -0.009 | 0.002 | 0.534 |
| Lt. pallidum | 0.000 | 0.000 | 0.000 | -0.008 | 0.000 | 0.173 |
| Lt. putamen | 0.002 | 0.001 | -0.001 | -0.007 | 0.001 | 0.973 |
| Lt. thalamus | 0.000 | 0.000 | 0.000 | -0.004 | 0.000 | 0.696 |
| Rt. accumbens | 0.007 | 0.000 | -0.007 | -0.006 | 0.000 | 0.036 |
| Rt. amygdala | 0.000 | 0.000 | 0.000 | -0.007 | 0.000 | 0.152 |
| Rt. caudate | 0.001 | 0.000 | -0.001 | -0.005 | 0.000 | 0.951 |
| Rt. hippocampus | 0.000 | 0.000 | 0.000 | -0.008 | 0.002 | 0.656 |
| Rt. pallidum | 0.001 | 0.000 | -0.001 | -0.009 | 0.000 | 0.686 |
| Rt. putamen | 0.000 | 0.000 | 0.000 | -0.007 | 0.001 | 0.315 |
| Rt. thalamus | 0.003 | 0.000 | -0.003 | -0.006 | 0.000 | 0.469 |
| Lt. angular | 0.000 | 0.000 | 0.000 | -0.004 | 0.000 | 0.140 |
| Lt. rolandic operculum | 0.001 | 0.000 | -0.001 | -0.009 | 0.001 | 0.584 |
| Lt. anterior cingulate and paracingulate | 0.001 | 0.001 | 0.000 | -0.010 | 0.001 | 0.250 |
| Lt. posterior cingulate | 0.007 | 0.000 | -0.007 | -0.009 | 0.002 | 0.076 |
| Lt. cuneus | 0.007 | 0.000 | -0.007 | -0.009 | 0.001 | 0.077 |
| Lt. frontal medial | 0.000 | 0.000 | 0.000 | -0.006 | 0.001 | 0.482 |
| Lt. frontal operculum | 0.000 | 0.001 | 0.001 | -0.007 | 0.001 | 0.152 |
| Lt. frontal orbital | 0.000 | 0.006 | 0.006 | -0.007 | 0.008 | 0.219 |
| Lt. frontal pole | 0.000 | 0.000 | 0.000 | -0.005 | 0.000 | 0.217 |
| Lt. heschls | 0.001 | 0.003 | 0.002 | -0.012 | 0.003 | 0.260 |
| Lt. inferior frontal pars opercularis | 0.000 | 0.005 | 0.005 | -0.010 | 0.005 | 0.207 |
| Lt. inferior frontal pars triangularis | 0.001 | 0.002 | 0.001 | -0.011 | 0.005 | 0.614 |
| Lt. inferior temporal anterior | 0.000 | 0.000 | 0.000 | -0.007 | 0.000 | 0.148 |
| Lt. inferior temporal posterior | 0.000 | 0.002 | 0.002 | -0.013 | 0.003 | 0.268 |
| Lt. inferior temporal temporooccipital | 0.000 | 0.000 | 0.000 | -0.009 | 0.001 | 0.455 |
| Lt. insular | 0.000 | 0.006 | 0.006 | -0.012 | 0.012 | 0.453 |
| Lt. intracalcarine | 0.000 | 0.000 | 0.000 | -0.008 | 0.001 | 0.667 |
| Lt. lateral occipital inferior | 0.000 | 0.003 | 0.003 | -0.009 | 0.001 | 0.097 |
| Lt. lateral occipital superior | 0.000 | 0.001 | 0.001 | -0.005 | 0.001 | 0.213 |
| Lt. lingual | 0.000 | 0.000 | 0.000 | -0.009 | 0.001 | 0.355 |
| Lt. middle frontal | 0.000 | 0.004 | 0.004 | -0.007 | 0.003 | 0.094 |
| Lt. middle temporal anterior | 0.000 | 0.000 | 0.000 | -0.011 | 0.002 | 0.419 |
| Lt. middle temporal posterior | 0.000 | 0.001 | 0.001 | -0.008 | 0.002 | 0.407 |
| Lt. middle temporal temporooccipital | 0.000 | 0.000 | 0.000 | -0.010 | 0.000 | 0.245 |
| Lt. occipital fusiform | 0.000 | 0.000 | 0.000 | -0.006 | 0.000 | 0.136 |
| Lt. occipital pole | 0.001 | 0.000 | -0.001 | -0.006 | 0.000 | 0.937 |
| Lt. paracingulate | 0.000 | 0.006 | 0.005 | -0.011 | 0.005 | 0.107 |
| Lt. parahippocampal anterior | 0.007 | 0.000 | -0.006 | -0.013 | 0.003 | 0.271 |
| Lt. parahippocampal posterior | 0.001 | 0.000 | -0.001 | -0.008 | 0.000 | 0.831 |
| Lt. parietal operculum | 0.001 | 0.000 | -0.001 | -0.005 | 0.000 | 0.981 |
| Lt. planum polare | 0.005 | 0.001 | -0.004 | -0.012 | 0.003 | 0.428 |
| Lt. planum temporale | 0.003 | 0.000 | -0.003 | -0.010 | 0.000 | 0.794 |
| Lt. postcentral | 0.001 | 0.001 | -0.001 | -0.006 | 0.000 | 0.757 |
| Lt. precentral | 0.000 | 0.001 | 0.001 | -0.008 | 0.002 | 0.354 |
| Lt. precuneuous | 0.000 | 0.003 | 0.003 | -0.007 | 0.005 | 0.272 |
| Lt. subcallosal | 0.000 | 0.000 | 0.000 | -0.009 | 0.000 | 0.254 |
| Lt. superior frontal | 0.000 | 0.002 | 0.002 | -0.007 | 0.001 | 0.080 |
| Lt. superior parietal | 0.000 | 0.000 | 0.000 | -0.008 | 0.000 | 0.620 |
| Lt. superior temporal anterior | 0.000 | 0.000 | 0.000 | -0.005 | 0.000 | 0.170 |
| Lt. superior temporal posterior | 0.000 | 0.000 | 0.000 | -0.005 | 0.000 | 0.142 |
| Lt. supracalcarine | 0.000 | 0.001 | 0.001 | -0.007 | 0.002 | 0.346 |
| Lt. supramarginal anterior | 0.001 | 0.000 | -0.001 | -0.005 | 0.000 | 0.946 |
| Lt. supramarginal posterior | 0.000 | 0.000 | 0.000 | -0.006 | 0.001 | 0.310 |
| Lt. temporal fusiform anterior | 0.005 | 0.000 | -0.004 | -0.011 | 0.001 | 0.439 |
| Lt. temporal fusiform posterior | 0.000 | 0.000 | 0.000 | -0.009 | 0.003 | 0.516 |
| Lt. temporal occipital fusiform | 0.000 | 0.001 | 0.001 | -0.009 | 0.001 | 0.221 |
| Lt. temporal pole | 0.000 | 0.000 | 0.000 | -0.011 | 0.001 | 0.312 |
| Rt. angular | 0.000 | 0.000 | 0.000 | -0.006 | 0.000 | 0.300 |
| Rt. rolandic operculum | 0.003 | 0.001 | -0.002 | -0.010 | 0.002 | 0.971 |
| Rt. anterior cingulate and paracingulate | 0.001 | 0.000 | -0.001 | -0.013 | 0.001 | 0.775 |
| Rt. posterior cingulate | 0.011 | 0.000 | -0.011 | -0.014 | 0.001 | 0.085 |
| Rt. cuneus | 0.000 | 0.000 | 0.000 | -0.008 | 0.000 | 0.670 |
| Rt. frontal medial | 0.000 | 0.001 | 0.000 | -0.009 | 0.001 | 0.298 |
| Rt. frontal operculum | 0.013 | 0.000 | -0.013 | -0.009 | 0.006 | 0.030 |
| Rt. frontal orbital | 0.000 | 0.000 | 0.000 | -0.008 | 0.001 | 0.267 |
| Rt. frontal pole | 0.000 | 0.000 | 0.000 | -0.004 | 0.001 | 0.388 |
| Rt. heschls | 0.002 | 0.000 | -0.002 | -0.012 | 0.001 | 0.845 |
| Rt. inferior frontal pars opercularis | 0.005 | 0.000 | -0.005 | -0.006 | 0.002 | 0.088 |
| Rt. inferior frontal pars triangularis | 0.019 | 0.000 | -0.019 | -0.012 | 0.008 | 0.018 |
| Rt. inferior temporal anterior | 0.003 | 0.000 | -0.003 | -0.012 | 0.002 | 0.882 |
| Rt. inferior temporal posterior | 0.000 | 0.001 | 0.000 | -0.010 | 0.005 | 0.675 |
| Rt. inferior temporal temporooccipital | 0.001 | 0.000 | -0.001 | -0.007 | 0.000 | 0.832 |
| Rt. insular | 0.009 | 0.007 | -0.001 | -0.010 | 0.028 | 0.335 |
| Rt. intracalcarine | 0.006 | 0.000 | -0.006 | -0.010 | 0.001 | 0.110 |
| Rt. lateral occipital inferior | 0.001 | 0.000 | -0.001 | -0.011 | 0.001 | 0.743 |
| Rt. lateral occipital superior | 0.000 | 0.000 | 0.000 | -0.006 | 0.000 | 0.514 |
| Rt. lingual | 0.002 | 0.001 | -0.002 | -0.010 | 0.001 | 0.933 |
| Rt. middle frontal | 0.001 | 0.003 | 0.002 | -0.007 | 0.003 | 0.286 |
| Rt. middle temporal anterior | 0.004 | 0.000 | -0.004 | -0.009 | 0.002 | 0.449 |
| Rt. middle temporal posterior | 0.001 | 0.008 | 0.007 | -0.011 | 0.010 | 0.211 |
| Rt. middle temporal temporooccipital | 0.000 | 0.000 | 0.000 | -0.007 | 0.001 | 0.289 |
| Rt. occipital fusiform | 0.000 | 0.001 | 0.001 | -0.010 | 0.001 | 0.237 |
| Rt. occipital pole | 0.000 | 0.001 | 0.001 | -0.007 | 0.000 | 0.131 |
| Rt. paracingulate | 0.000 | 0.006 | 0.006 | -0.012 | 0.005 | 0.104 |
| Rt. parahippocampal anterior | 0.001 | 0.000 | 0.000 | -0.009 | 0.000 | 0.522 |
| Rt. parahippocampal posterior | 0.000 | 0.000 | 0.000 | -0.007 | 0.000 | 0.175 |
| Rt. parietal operculum | 0.000 | 0.000 | 0.000 | -0.008 | 0.001 | 0.343 |
| Rt. planum polare | 0.001 | 0.000 | -0.001 | -0.013 | 0.002 | 0.733 |
| Rt. planum temporale | 0.002 | 0.000 | -0.002 | -0.010 | 0.001 | 0.936 |
| Rt. postcentral | 0.001 | 0.001 | 0.000 | -0.007 | 0.002 | 0.720 |
| Rt. precentral | 0.001 | 0.007 | 0.006 | -0.007 | 0.007 | 0.189 |
| Rt. precuneus | 0.000 | 0.001 | 0.000 | -0.007 | 0.002 | 0.565 |
| Rt. subcallosal | 0.000 | 0.000 | 0.000 | -0.007 | 0.000 | 0.215 |
| Rt. superior frontal | 0.000 | 0.002 | 0.002 | -0.014 | 0.001 | 0.143 |
| Rt. superior parietal | 0.000 | 0.000 | 0.000 | -0.005 | 0.000 | 0.548 |
| Rt. superior temporal anterior | 0.000 | 0.000 | 0.000 | -0.007 | 0.000 | 0.175 |
| Rt. superior temporal posterior | 0.001 | 0.000 | -0.001 | -0.011 | 0.000 | 0.808 |
| Rt. supracalcarine | 0.000 | 0.000 | 0.000 | -0.008 | 0.001 | 0.250 |
| Rt. supramargianl anterior | 0.011 | 0.000 | -0.011 | -0.006 | 0.000 | 0.010 |
| Rt. supramargianl posterior | 0.001 | 0.000 | -0.001 | -0.007 | 0.000 | 0.883 |
| Rt. temporal fusiform anterior | 0.000 | 0.000 | 0.000 | -0.005 | 0.000 | 0.197 |
| Rt. temporal fusiform posterior | 0.000 | 0.001 | 0.001 | -0.011 | 0.005 | 0.568 |
| Rt. temporal occipital fusiform | 0.001 | 0.000 | -0.001 | -0.007 | 0.001 | 0.734 |
| Rt. temporal pole | 0.002 | 0.001 | -0.001 | -0.010 | 0.002 | 0.836 |
|  | TLE patients with HS | TLE patients without HS | Difference | CI lower | CI upper | *p*-value |
| Brainstem | 0.000 | 0.010 | 0.010 | -0.006 | 0.010 | 0.080 |
| Lt. accumbens | 0.001 | 0.000 | -0.001 | -0.003 | 0.008 | 0.484 |
| Lt. amygdala | 0.001 | 0.000 | -0.001 | -0.005 | 0.012 | 0.441 |
| Lt. caudate | 0.007 | 0.000 | -0.007 | -0.006 | 0.010 | 0.083 |
| Lt. hippocampus | 0.000 | 0.000 | 0.000 | 0.000 | 0.003 | 0.240 |
| Lt. pallidum | 0.002 | 0.000 | -0.002 | -0.003 | 0.010 | 0.209 |
| Lt. putamen | 0.005 | 0.002 | -0.003 | -0.006 | 0.009 | 0.310 |
| Lt. thalamus | 0.004 | 0.000 | -0.004 | -0.009 | 0.012 | 0.329 |
| Rt. accumbens | 0.000 | 0.007 | 0.007 | -0.003 | 0.008 | 0.070 |
| Rt. amygdala | 0.001 | 0.000 | -0.001 | -0.001 | 0.005 | 0.228 |
| Rt. caudate | 0.002 | 0.001 | -0.001 | -0.001 | 0.005 | 0.158 |
| Rt. hippocampus | 0.000 | 0.000 | 0.000 | -0.001 | 0.003 | 0.353 |
| Rt. pallidum | 0.000 | 0.001 | 0.001 | -0.001 | 0.006 | 0.833 |
| Rt. putamen | 0.001 | 0.000 | -0.001 | -0.008 | 0.011 | 0.491 |
| Rt. thalamus | 0.002 | 0.003 | 0.001 | -0.003 | 0.007 | 0.883 |
| Lt. angular | 0.000 | 0.000 | 0.000 | 0.000 | 0.002 | 0.309 |
| Lt. rolandic operculum | 0.005 | 0.001 | -0.004 | -0.011 | 0.013 | 0.497 |
| Lt. anterior cingulate and paracingulate | 0.000 | 0.001 | 0.001 | -0.016 | 0.028 | 0.813 |
| Lt. posterior cingulate | 0.000 | 0.007 | 0.007 | -0.003 | 0.006 | 0.039 |
| Lt. cuneus | 0.000 | 0.007 | 0.007 | -0.004 | 0.010 | 0.151 |
| Lt. frontal medial | 0.006 | 0.000 | -0.006 | -0.004 | 0.008 | 0.088 |
| Lt. frontal operculum | 0.008 | 0.000 | -0.008 | -0.008 | 0.013 | 0.119 |
| Lt. frontal orbital | 0.001 | 0.000 | -0.001 | -0.001 | 0.004 | 0.158 |
| Lt. frontal pole | 0.004 | 0.000 | -0.004 | -0.003 | 0.005 | 0.077 |
| Lt. heschls | 0.008 | 0.001 | -0.007 | -0.009 | 0.013 | 0.202 |
| Lt. inferior frontal pars opercularis | 0.003 | 0.000 | -0.003 | -0.007 | 0.009 | 0.479 |
| Lt. inferior frontal pars triangularis | 0.000 | 0.001 | 0.001 | -0.002 | 0.006 | 0.992 |
| Lt. inferior temporal anterior | 0.000 | 0.000 | 0.000 | -0.002 | 0.004 | 0.495 |
| Lt. inferior temporal posterior | 0.000 | 0.000 | 0.000 | -0.001 | 0.003 | 0.397 |
| Lt. inferior temporal temporooccipital | 0.000 | 0.000 | 0.000 | -0.003 | 0.009 | 0.702 |
| Lt. insular | 0.005 | 0.000 | -0.005 | -0.011 | 0.011 | 0.504 |
| Lt. intracalcarine | 0.000 | 0.000 | 0.000 | -0.001 | 0.005 | 0.869 |
| Lt. lateral occipital inferior | 0.000 | 0.000 | 0.000 | -0.006 | 0.008 | 0.813 |
| Lt. lateral occipital superior | 0.001 | 0.000 | -0.001 | -0.003 | 0.006 | 0.398 |
| Lt. lingual | 0.001 | 0.000 | -0.001 | -0.005 | 0.010 | 0.588 |
| Lt. middle frontal | 0.003 | 0.000 | -0.003 | -0.003 | 0.006 | 0.109 |
| Lt. middle temporal anterior | 0.000 | 0.000 | 0.000 | -0.002 | 0.005 | 0.393 |
| Lt. middle temporal posterior | 0.000 | 0.000 | 0.000 | -0.001 | 0.003 | 0.508 |
| Lt. middle temporal temporooccipital | 0.000 | 0.000 | 0.000 | -0.006 | 0.012 | 0.508 |
| Lt. occipital fusiform | 0.003 | 0.000 | -0.003 | -0.004 | 0.009 | 0.168 |
| Lt. occipital pole | 0.000 | 0.001 | 0.001 | -0.001 | 0.004 | 0.356 |
| Lt. paracingulate | 0.014 | 0.000 | -0.013 | -0.014 | 0.017 | 0.125 |
| Lt. parahippocampal anterior | 0.001 | 0.007 | 0.006 | -0.007 | 0.012 | 0.294 |
| Lt. parahippocampal posterior | 0.000 | 0.001 | 0.001 | -0.003 | 0.007 | 0.956 |
| Lt. parietal operculum | 0.000 | 0.001 | 0.001 | -0.003 | 0.005 | 0.971 |
| Lt. planum polare | 0.006 | 0.005 | -0.001 | -0.009 | 0.011 | 0.837 |
| Lt. planum temporale | 0.000 | 0.003 | 0.003 | -0.002 | 0.006 | 0.302 |
| Lt. postcentral | 0.009 | 0.001 | -0.008 | -0.009 | 0.011 | 0.156 |
| Lt. precentral | 0.011 | 0.000 | -0.011 | -0.009 | 0.013 | 0.078 |
| Lt. precuneuous | 0.003 | 0.000 | -0.003 | -0.010 | 0.009 | 0.650 |
| Lt. subcallosal | 0.006 | 0.000 | -0.006 | -0.005 | 0.011 | 0.096 |
| Lt. superior frontal | 0.006 | 0.000 | -0.006 | -0.006 | 0.012 | 0.119 |
| Lt. superior parietal | 0.002 | 0.000 | -0.002 | -0.005 | 0.007 | 0.425 |
| Lt. superior temporal anterior | 0.000 | 0.000 | 0.000 | -0.003 | 0.005 | 0.504 |
| Lt. superior temporal posterior | 0.000 | 0.000 | 0.000 | -0.001 | 0.004 | 0.331 |
| Lt. supracalcarine | 0.001 | 0.000 | -0.001 | -0.002 | 0.005 | 0.313 |
| Lt. supramarginal anterior | 0.005 | 0.001 | -0.004 | -0.006 | 0.012 | 0.216 |
| Lt. supramarginal posterior | 0.000 | 0.000 | 0.000 | -0.002 | 0.006 | 0.483 |
| Lt. temporal fusiform anterior | 0.001 | 0.005 | 0.003 | -0.006 | 0.009 | 0.421 |
| Lt. temporal fusiform posterior | 0.001 | 0.000 | -0.001 | -0.006 | 0.010 | 0.504 |
| Lt. temporal occipital fusiform | 0.001 | 0.000 | -0.001 | -0.003 | 0.007 | 0.249 |
| Lt. temporal pole | 0.000 | 0.000 | 0.000 | -0.002 | 0.006 | 0.506 |
| Rt. angular | 0.001 | 0.000 | -0.001 | -0.004 | 0.007 | 0.376 |
| Rt. rolandic operculum | 0.001 | 0.003 | 0.002 | -0.004 | 0.009 | 0.902 |
| Rt. anterior cingulate and paracingulate | 0.001 | 0.001 | 0.001 | -0.017 | 0.020 | 0.949 |
| Rt. posterior cingulate | 0.000 | 0.011 | 0.011 | -0.005 | 0.009 | 0.047 |
| Rt. cuneus | 0.001 | 0.000 | 0.000 | -0.004 | 0.009 | 0.670 |
| Rt. frontal medial | 0.005 | 0.000 | -0.004 | -0.002 | 0.006 | 0.062 |
| Rt. frontal operculum | 0.001 | 0.013 | 0.012 | -0.005 | 0.008 | 0.020 |
| Rt. frontal orbital | 0.003 | 0.000 | -0.003 | -0.004 | 0.006 | 0.212 |
| Rt. frontal pole | 0.003 | 0.000 | -0.003 | -0.003 | 0.005 | 0.155 |
| Rt. heschls | 0.006 | 0.002 | -0.005 | -0.013 | 0.018 | 0.485 |
| Rt. inferior frontal pars opercularis | 0.001 | 0.005 | 0.004 | -0.002 | 0.003 | 0.054 |
| Rt. inferior frontal pars triangularis | 0.000 | 0.019 | 0.019 | -0.003 | 0.008 | 0.004 |
| Rt. inferior temporal anterior | 0.004 | 0.003 | 0.000 | -0.014 | 0.014 | 0.988 |
| Rt. inferior temporal posterior | 0.000 | 0.000 | 0.000 | -0.003 | 0.004 | 0.856 |
| Rt. inferior temporal temporooccipital | 0.001 | 0.001 | -0.001 | -0.006 | 0.009 | 0.686 |
| Rt. insular | 0.012 | 0.009 | -0.004 | -0.024 | 0.018 | 0.963 |
| Rt. intracalcarine | 0.000 | 0.006 | 0.006 | -0.002 | 0.007 | 0.063 |
| Rt. lateral occipital inferior | 0.018 | 0.001 | -0.017 | -0.026 | 0.020 | 0.267 |
| Rt. lateral occipital superior | 0.010 | 0.000 | -0.010 | -0.008 | 0.013 | 0.087 |
| Rt. lingual | 0.001 | 0.002 | 0.001 | -0.023 | 0.024 | 0.890 |
| Rt. middle frontal | 0.000 | 0.001 | 0.001 | -0.001 | 0.003 | 0.246 |
| Rt. middle temporal anterior | 0.000 | 0.004 | 0.004 | -0.002 | 0.005 | 0.153 |
| Rt. middle temporal posterior | 0.002 | 0.001 | -0.001 | -0.005 | 0.005 | 0.777 |
| Rt. middle temporal temporooccipital | 0.001 | 0.000 | -0.001 | -0.003 | 0.006 | 0.279 |
| Rt. occipital fusiform | 0.013 | 0.000 | -0.013 | -0.010 | 0.015 | 0.069 |
| Rt. occipital pole | 0.001 | 0.000 | -0.001 | -0.003 | 0.006 | 0.340 |
| Rt. paracingulate | 0.001 | 0.000 | -0.001 | -0.002 | 0.009 | 0.289 |
| Rt. parahippocampal anterior | 0.000 | 0.001 | 0.001 | -0.001 | 0.004 | 0.953 |
| Rt. parahippocampal posterior | 0.000 | 0.000 | 0.000 | -0.001 | 0.002 | 0.368 |
| Rt. parietal operculum | 0.000 | 0.000 | 0.000 | -0.001 | 0.004 | 0.231 |
| Rt. planum polare | 0.009 | 0.001 | -0.007 | -0.014 | 0.017 | 0.321 |
| Rt. planum temporale | 0.000 | 0.002 | 0.002 | -0.005 | 0.010 | 0.657 |
| Rt. postcentral | 0.011 | 0.001 | -0.010 | -0.010 | 0.011 | 0.106 |
| Rt. precentral | 0.004 | 0.001 | -0.003 | -0.007 | 0.009 | 0.417 |
| Rt. precuneus | 0.012 | 0.000 | -0.012 | -0.007 | 0.008 | 0.029 |
| Rt. subcallosal | 0.008 | 0.000 | -0.008 | -0.008 | 0.009 | 0.113 |
| Rt. superior frontal | 0.003 | 0.000 | -0.003 | -0.002 | 0.006 | 0.084 |
| Rt. superior parietal | 0.000 | 0.000 | 0.000 | -0.006 | 0.009 | 0.707 |
| Rt. superior temporal anterior | 0.001 | 0.000 | -0.001 | -0.001 | 0.004 | 0.236 |
| Rt. superior temporal posterior | 0.000 | 0.001 | 0.001 | -0.004 | 0.008 | 0.915 |
| Rt. supracalcarine | 0.000 | 0.000 | 0.000 | -0.002 | 0.005 | 0.517 |
| Rt. supramarginal anterior | 0.000 | 0.011 | 0.011 | -0.002 | 0.007 | 0.026 |
| Rt. supramarginal posterior | 0.000 | 0.001 | 0.001 | -0.001 | 0.006 | 0.948 |
| Rt. temporal fusiform anterior | 0.001 | 0.000 | -0.001 | -0.002 | 0.005 | 0.260 |
| Rt. temporal fusiform posterior | 0.002 | 0.000 | -0.002 | -0.005 | 0.006 | 0.275 |
| Rt. temporal occipital fusiform | 0.015 | 0.001 | -0.014 | -0.018 | 0.018 | 0.239 |
| Rt. temporal pole | 0.006 | 0.002 | -0.004 | -0.012 | 0.010 | 0.724 |

TLE: temporal lobe epilepsy, HS: hippocampal sclerosis, CI: 95% confidence interval
